# Distinct patterns of cortical atrophy characterize AD pathology

**DOI:** 10.1002/alz70857_106962

**Published:** 2025-12-26

**Authors:** Panagiotis Georgios Passias, Roberto Vicidomini, Rifa Sanjida Punnota, Gayatri Kulkarni, Paul Edison, Alzheimer's Disease Neuroimaging Initiative (ADNI)

**Affiliations:** ^1^ Imperial College London, London, Greater London, United Kingdom

## Abstract

**Background:**

Medial Temporal Lobe (MTL) has traditionally been considered a key region of neurodegeneration in Alzheimer's Disease (AD). However, a significant proportion of patients present with cognitive impairment without involvement of this area. The pattern of cortical degeneration in these groups remains unclear. In this study, we evaluated whether regional atrophy could begin in other regions different from MTL in the AD trajectory.

**Method:**

1124 participants were selected from the ADNI database. Region of Interest analysis ‐ derived volumes as well as Cerebrospinal Fluid (CSF) biomarkers data were collected, including Aβ42, Aβ40, total tau and phosphorylated tau. Standardized Uptake Value Ratios were generated in SPM for Tau, Amyloid and FDG PET images. To detect the earliest changes, we evaluated lobar volumes that were more than one standard deviation below the mean of amyloid‐negative controls. Subjects with isolated frontal or occipital atrophy were excluded from the results.

**Result:**

In the whole population, 25% of patients belonged to the parietal atrophy subgroup, of whom 46% were Amyloid‐positive on PET compared to 64% of the Medial Temporal Group. The proportion of Parietal dominant variant was higher in early stages of the disease (SMC: 43%, EMCI: 31%, LMCI: 18%, AD: 8%). ADAS‐Cog‐13 scores were lower in the Parietal Group, while MMSE scores were higher. Evaluation of CSF biomarkers showed a reduced ptau/tau and ptau/Aβ42 ratios but no difference in the Aβ42/Aβ40 ratio in the parietal group relative to the Medial Temporal Group. Parietal Group was characterized by higher Amyloid deposition in the hippocampus and lower Tau levels in the MTL compared to the Medial Temporal Group.

**Conclusion:**

A significant percentage (25%) of patients with memory impairment present with isolated Parietal Atrophy which displays a distinctive set of biological, imaging and clinical features. Lower Tau accumulation in the Parietal variant may underlie its relatively milder cognitive impairment, reinforcing the role of Tau as the primary driver of phenotypic differences between the two groups. This could be highly relevant in clinical practice to ensure precise subcategorization of patients with cognitive impairment, addressing them to the most appropriate targeted therapy.

